# QuantiFERON-TB Gold In-Tube test conversions and reversions among tuberculosis patients and their household contacts in Addis Ababa: a one year follow-up study

**DOI:** 10.1186/s12879-014-0654-5

**Published:** 2014-12-03

**Authors:** Mulugeta Belay, Mengistu Legesse, Daniel Dagne, Adane Mihret, Yonas Bekele, Girmay Medhin, Gunnar Bjune, Fekadu Abebe

**Affiliations:** Aklilu Lemma Institute of Pathobiology, Addis Ababa University, Addis Ababa, Ethiopia; Department of Community Medicine, Institute of Health and Society, University of Oslo, Blindern, 0318 Oslo, Norway; Dessie Regional Health Research Laboratory Center, Amhara Regional Health Bureau, Dessie, Ethiopia; Armauer Hansen Research Institute, Addis Ababa, Ethiopia

**Keywords:** Tuberculosis, QuantiFERON-TB-Gold In-Tube, Conversion, Reversion, Patients, Contacts, Ethiopia

## Abstract

**Background:**

QuantiFERON-TB Gold In-Tube® (QFT-GIT) test is used for the diagnosis of latent tuberculosis (TB) infection. Besides, QFT-GIT test could allow tracking changes in immune response among TB patients and their contacts. In high TB burden settings, reports on QFT-GIT conversions and reversions among TB patients and their contacts are limited. As part of a major project to study immune responses to TB infection, we investigated QFT-GIT test conversions and reversions among smear positive pulmonary TB patients and their household contacts over 12 months.

**Methods:**

We followed a total of 107 HIV negative participants (33 patients and 74 contacts) in Addis Ababa. We did QFT-GIT test at baseline and 12 months later according to the manufacturer’s instructions.

**Results:**

At baseline, 25/33 (75.8%) of the patients and 50/74 (67.6%) of the contacts were QFT-GIT positive. At 12 months, 2 more patients (1 test negative and 1 indeterminate) became test positive. Besides, 11/24 (45.8%) test negative contacts became positive. Only one patient and one contact who were test positive at baseline became test negative 12 months later. At 12 months, the proportions of QFT-GIT test positives for patients and contacts were, therefore, 78.8% and 81.1%, respectively. Among contacts, the proportion of QFT-GIT test positives at 12 months was significantly higher compared to the corresponding proportion at baseline (McNemar, p = 0.006); similarly, the median IFN-γ response significantly increased at 12 months compared with the baseline level (Wilcoxon matched-pairs signed rank test, p = 0.01). Patients, however, had comparable median IFN-γ levels at baseline and 12 months later (p = 0.56).

**Conclusion:**

Nearly half of QFT-GIT negative household contacts at baseline became positive at 12 months. This suggests that repeated screening of QFT-GIT negative contacts may be needed for epidemiological studies and interventions of latent TB in an endemic setting. A large longitudinal study may be needed to confirm our observations.

**Electronic supplementary material:**

The online version of this article (doi:10.1186/s12879-014-0654-5) contains supplementary material, which is available to authorized users.

## Background

Tuberculosis (TB) remains a serious public health problem, especially in low income countries, despite the global effort to tackle it for the past 20 years. In 2012 alone, there were 8.6 million incident cases and 1.3 million deaths [1]. Early detection and proper treatment of TB cases are the main strategies to control TB [2]. However, delayed diagnosis of TB patients and transmission to contacts is a major challenge. Although infection rates are high, the majority of infected contacts do not progress to active TB. The risk of progression to active TB is greatest in the first 2-5 years following infection [3],[4].

Preventive therapy among household contacts of TB patients has been the practice in most low prevalence countries as one of the key strategies to eliminate TB. However, in TB endemic countries, the prevalence of latent TB is high and preventive therapy is limited to those individuals with HIV infection and children <5 years of age with an adult household contact [5]. Tuberculin skin test (TST) has been routinely used to screen latent TB; however, it has poor specificity among BCG vaccinated populations [6],[7]. Currently, two T-cell based interferon gamma release assays (IGRAs) are being used for the diagnosis of latent TB infection and surveillance of new infections. The high specificity of IGRAs to TB infection even among BCG vaccinated population [7],[8] makes them more appealing.

QuantiFERON-TB Gold In-Tube® (QFT-GIT) test is one of the two commercialized IGRA tests being used for the diagnosis of latent TB infection. Conversions and reversions are reported to be common in studies from low TB prevalence countries [9]–[11]. Conversions indicate recent infections with increased risk of progression [12] whereas reversions may indicate bacterial clearance. There is limited information regarding conversions and reversions of QFT-GIT test results among pulmonary TB patients and their household contacts in high TB burden countries. Here, for the first time in Africa, we report conversions and reversions among TB patients and their household contacts over 12 months.

## Methods

### Study setting and population

This study was conducted as part of a large follow-up study investigating the role of humoral and cell mediated immune protection during *M. tuberculosis* infection among a human population in Addis Ababa. Addis Ababa has a population of 2.6 million [13].

TB patients are primarily treated at health centers. Out of 24 health centers with established Directly Observed Treatment, Short course (DOTS) services, 7 health centers (Kotebe, Akaki, Kazanchis, Bole, Shiro Meda, Woreda 7 and Teklehaymanot) were selected for the study. From April to December 2012, smear positive pulmonary TB patients were recruited before treatment initiation. The diagnosis of smear positive pulmonary TB was made when at least two out of three consecutive sputum smear examinations were positive for acid fast bacilli. Subsequently, successful growth on Lowenstein-Jensen solid media was used to confirm diagnosis. Patients were treated with anti-TB drugs for 6 months according to the national guideline [5]. Household contacts living in the same house with smear positive TB patients were screened for TB using clinical assessment and chest x-ray. AFB and culture was done for those with productive cough. Contacts donated blood samples within 1 to 7 days following diagnosis of TB among patients. No prophylactic treatment was given to contacts without evidence for TB. Patients were assessed for any concurrent illnesses including medication side effects. Contacts were reassessed at 6 months and were also encouraged to report any illness between follow-up visits. All participants were adults (18-60 years). HIV testing was done according to the national guideline [14] and only HIV negative patients and contacts were included.

### Sample collection and ELISA

QFT-GIT test was performed according to the manufacturer’s instructions (Cellestis, Carnegie). Briefly, 1 ml of whole blood was drawn into each of the three tubes supplied by the manufacturer. Each tube contained no antigen (negative control), PHA (positive control) or peptides of the *M. tuberculosis* antigens Early Secretory Antigenic Target 6 (ESAT-6), Culture Filtrate Protein 10 (CFP-10) and TB 7.7 (p4). Blood specimens from all health centers were transported and incubated at Armauer Hansen Research Institute (AHRI) laboratory within 4-6 hours of collection. The tubes were incubated for 24 hours at 37°C, centrifuged at 3000 rpm for 15 minutes and supernatants stored at -80°C until enzyme-linked immunosorbent assay was done to measure IFN-γ production. Calculation of results was done using QuantiFERON ®-TB Gold analysis software version 2.62 (Cellestis, Carnegie, Australia). A positive test result was determined at a cut-off value of 0.35 IU/ml, according to the manufacturer’s recommendation. Standard operating procedures as well as company’s instructions were followed during sample collection, incubation, storage and IFN-γ measurement to minimize potential sources of variability.

### Data analysis

QFT-GIT test positive proportions were calculated. McNemar test was used to compare test positive proportions at baseline and 12 months later. Mann-Whitney test was used to compare IFN-γ response among contacts and patients. Wilcoxon matched-pairs signed rank test was used to investigate changes in IFN-γ levels at 12 months compared to baseline levels. GraphPad Prism version 6 (GraphPad Software, La Jolla California USA, http://www.graphpad.com) was used to analyze the data. A p-value less than 0.05 was considered statistically significant.

### Ethics statement

The study protocol was approved by the Institutional Review Board of Aklilu Lemma Institute of Pathobiology, AHRI/ALERT Ethics Review Committee, The National Health Research Ethics Review Committee from Ethiopia and the Regional Committees for Medical Research Ethics, South East Norway (Regionale komiteer for medisinsk og helsefaglig forskningsetikk sør-øst) from Norway. Written informed consent was obtained from each participant.

## Results

A total of 107 HIV negative participants (33 smear positive pulmonary TB patients and 74 household contacts) were followed over one year. The median age was 28 years (IQR: 23-42 years). Patients (median age of 27 years) and contacts (median age of 29 years) had comparable median age. Overall, females constituted 55.7% of the study participants. There were more females among contacts (60.8%) compared to patients (43.8%). However, the difference was not statistically significant (X^2^ = 2.6, p = 0.11).

The median duration of cough among patients before treatment was 8 weeks (IQR: 4-29 weeks). Among contacts, 27 (36.5%) reported sleeping on the same bed and 18 (24.3%) in the same room with the patient. The rest were sleeping in a different room in the same house. No significant difference was observed between proximity of sleeping and infection. On average, contacts reported a median contact duration of 12 hours (IQR: 9-12 hours) per day with the patient.

At 6 months, all patients were declared cured based on sputum conversion and clinical assessment. Among household contacts, two progressed to active TB: one of these contacts was diagnosed as smear positive at 6 months and the other contact was diagnosed as smear negative and culture positive at 12 months. At baseline, 25 (75.8%) of the patients were QFT-GIT positive, one was indeterminate and 7 were QFT-GIT negative. Similarly, 50 (67.6%) of the contacts were QFT-GIT positive and the rest were QFT-GIT negative.

With regard to conversions and reversions, after 12 months, only one patient with a negative QFT-GIT test result and another patient with indeterminate result became positive. Besides, only 1/25 (4%) QFT-GIT positive patients at baseline became QFT-GIT negative at 12 months. Among contacts, 11/24 (45.8%) of QFT-GIT negative participants became positive. On the other hand, only 1/50 (2%) QFT-GIT positive contacts became negative. In total, 60 (81.1%) of the contacts were QFT-GIT positive at 12 months. The two contacts who progressed to active TB were QFT-GIT negative at baseline but both were QFT-GIT positive at diagnosis of active TB.

**Figure 1:**
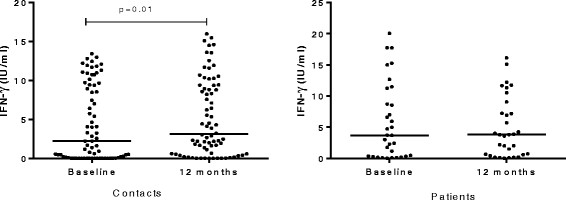
**Comparison of median levels of IFN-γ at baseline and 12 months later among patients (n = 32) and contacts (n = 74).** Wilcoxon matched-pairs signed rank test was used to analyze the data. Each filled circle represents a participant and the horizontal solid lines represent medians. The median level of IFN-γ among contacts was significantly increased at 12 months compared to baseline level but among patients, there was no significant difference.

**Figure 2:**
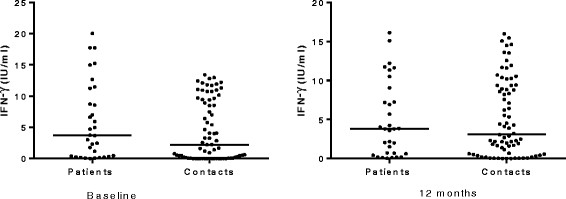
**Comparison of the median levels of IFN-γ between patients (n = 32) and contacts (n = 74) at baseline and 12 months.** Mann-Whitney test was used to analyze the data. Each filled circle represents a participant and horizontal solid lines represent medians. There was no statistically significant difference in the median levels of IFN-γ between patients and contacts both at baseline and 12 months later.

Among household contacts, the proportion of QFT-GIT positive was significantly higher at 12 months compared to the proportion at baseline (McNemar, p = 0.006); however, no significant difference was observed among patients (McNemar, p = 1.00) (Table 1). Among household contacts, the median level of IFN-γ significantly increased at 12 months compared to baseline levels (Wilcoxon matched pairs signed rank test, p = 0.01). Patients had comparable median IFN-γ levels at baseline and 12 months later (p = 0.56) (Figure 1). With regard to the proportion of QFT-GIT positives, there was no statistically significant difference between contacts and patients both at baseline (Mann-Whitney, p = 0.35) and 12 months later (Mann-Whitney, p = 0.98). Similarly, the median levels of IFN-γ among patients and contacts were comparable both at baseline and 12 months later (Figure 2). Among 11 contacts with conversions, 9 had IFN-γ level above 1 IU/ml whereas only 2 had just above 0.5 IU/ml indicating a strong IFN-γ response among converters (Table 2, Additional file 1).

**Table 1:** **Comparison of QFT-GIT test results at baseline and 12 months among contacts and patients**

	Patients		Contacts	
	n(%)		n(%)	
	Baseline	12 months	Baseline	12 months
QFT-GIT-negative	7 (21.9*)	6 (18.8*)	24 (32.4)	14 (18.9)
QFT-GIT-positive	25 (78.1*)	26 (81.2*)	50 (67.6)	^**†**^60(81.1)
McNemar, p-value	1.00		0.006	

*One patient with indeterminate result at baseline was excluded from the denominator.

^**†**^One QFT-GIT positive contact at baseline became negative at 12 months and hence the total QFT-GIT positive contacts at 12 months were 60 instead of 61 (50 + 11).

**Table 2:** **Levels of IFN-γ at baseline and 12 months later among QFT-GIT negative contacts at baseline**

Participant	IFN-γ (IU/ml)	IFN-γ (IU/ml)	QFT-GIT result at 12 months
	at baseline	at 12 months	
**Non-converters**			
1	0.00	0.05	Negative
2	0.02	0.02	Negative
3	0.12	0.21	Negative
4	0.03	0.03	Negative
5	0.00	0.34	Negative
6	0.00	0.00	Negative
7	0.00	0.04	Negative
8	0.00	0.08	Negative
9	0.00	0.03	Negative
10	0.06	0.17	Negative
11	0.00	0.00	Negative
12	0.01	0.00	Negative
13	0.00	0.03	Negative
**Converters**			
14	0.19	0.53	Positive
15	0.28	0.57	Positive
16	0.21	1.15	Positive
17	0.00	2.19	Positive
18	0.02	2.27	Positive
19	0.03	2.47	Positive
20	0.01	2.68	Positive
21	0.08	4.31	Positive
22	0.02	4.52	Positive
23	0.04	5.54	Positive
24	0.00	8.94	Positive

Contacts from number 1 to 13 (n = 13) were QFT-GIT negative both at baseline and at 12 months with low IFN-γ response whereas contacts from number 14 to 24 (n = 11) were negative at baseline but became positive at 12 months. Nine of the 11 contacts with QFT-GIT test conversions had high IFN-γ response at 12 months whereas 2 (number 14 and 15) of them had IFN-γ values close to the cut-off (0.35).

## Discussion

The use of QFT-GIT test in longitudinal studies could allow tracking changes in immune response over time. In this study, we investigated conversions and reversions of QFT-GIT test results among TB patients and their household contacts in a high TB burden setting. The key findings of this study included: (a) a very low rate of reversion both in patients and contacts, and (b) a high rate of conversion among household contacts.

At baseline, a very high proportion of contacts were QFT-GIT positive in this study. However, in other high burden settings, similar studies [15],[16] reported a lower prevalence (54-59%) of latent TB infection among household contacts. This difference might be attributable to the differences in the degree of exposure. Previously, a 63.7% prevalence of latent TB infection was diagnosed among healthy adult pastoralists in Afar Region [17] suggesting that there is already a high rate of latent TB infection among the population. Besides, a long diagnostic delay among TB patients is a major problem in Ethiopia [18] and infection from recent exposure might account for the high prevalence of latent TB among household contacts in the study area.

Previous studies reported different rates of QFT-GIT test conversions and reversions among household contacts and those with no obvious contact history. Comparable to our finding, a study from Uganda reported a TST conversion rate of 41% among household contacts over a year [19]. Similarly, another study from India reported a higher QFT-GIT test conversion rate compared to reversions [16]; however, they also reported a high reversion rate unlike our findings. Contrary to our findings, other studies reported that reversions were found to be more common compared to conversions [10],[20]. For example, a study from the US among HIV infected individuals whose contact history was unknown reported that a third of QFT-GIT test positive individuals became negative at 24 months and the conversion rate was only 9% [20]. Another study among German health care workers reported a low rate of conversion (1.9%) compared to the rate of reversion (33.3%) [10]. Unlike the participants in these two studies, our study participants were household contacts of smear positive pulmonary TB patients and some of them could be in the incubation period at baseline with subsequent test conversions. Besides, in Ethiopia where the estimated prevalence of smear positive pulmonary TB is 108 per 100,000 population [21], multiple exposures to pulmonary TB patients are expected and during the follow-up period, some of our contacts might have been exposed out of home and infected. This could partly explain the high conversion and the low reversion rates. Other factors including geographical [22] and *M.tuberculosis* strain [23] variations are also reported to be associated with variations in IFN-γ response and hence may partly explain the difference observed across settings.

In pulmonary TB patients, reversions and conversions were very low. Besides, the baseline and 12 months median levels of IFN-γ were comparable. However, studies from The Gambia [24], Japan [25] and South Korea [26] reported high reversion rates following treatment completion. Reasons for such variations are not clear from the current study. However, in high burden settings like Ethiopia, IFN-γ could possibly be boosted from casual contact with other TB patients. Supporting this assertion is the fact that during the 6 month treatment period, TB patients come in contact with each other without restriction at DOTS clinics. Besides, the low reversion rate in our study could be due to stimulation of the immune system by persistent *M.tuberculosis* infection as suggested previously [27]. Although the sample size is small, our result confirms that QFT-GIT may not be useful for treatment monitoring among TB patients in high burden setting as previously reported [28].

In our study, 2 household contacts who were QFT-GIT test negative at baseline progressed to active pulmonary TB. Although the data is small, this contradicts the suggestion that IFN-γ release assays could potentially identify those with a high risk of progression among latently infected individuals [16]. Converters could be important groups in terms of disease progression. For example, a study from South Africa compared the risk of progression between converters and persistent QFT-GIT negative adolescents and found a significantly increased risk of progression among converters. Our finding suggests the need for repeated screening of QFT-GIT negatives especially if prophylactic anti-TB treatment is offered for latent TB infection based on QFT-GIT test result.

This study has revealed important findings. However, the results of this study need to be interpreted in view of its limitations. We were not able to compare QFT-GIT test results with TST results since we deliberately omitted TST for the larger cohort to avoid possible sensitization of immune responses. However, we believe this may not affect our findings as many studies have reported a good degree of agreement between these two tests and QFT-GIT is more specific to TB infection compared to TST. QFT-GIT test variability could be a potential source of bias but we strictly followed the company’s recommendation during sample collection, incubation, sample storage and ELISA and hence the impact of such variability is expected to be minimal. Almost all converters had IFN-γ levels much higher than the cut-off value and hence they are most likely true converters. The other limitation of this study is the exclusion of children as well as those with immunosuppressive conditions and therefore, the findings of this study may not be generalized to these groups.

## Conclusion

Conversions are common among household contacts in this study; however, reversions were rare both among patients and contacts. Therefore, for epidemiologic studies and interventions of latent TB, repeated screening of QFT-GIT negative contacts may be needed to diagnose TB infection in a high TB burden setting. A larger follow-up study including children and those with immunosuppressive conditions could generate more evidence on the rate of conversions and reversions as well as the risk of progression to active TB among different groups (converters, persistent QFT-GIT positives and persistent QFT-GIT negatives).

## Additional file

## Electronic supplementary material

Additional file 1: Levels of IFN-γ at baseline and 12 months later among 24 contacts with baseline QFT-GIT negative results. Wilcoxon matched-pairs signed rank test was used to analyze the data. Each filled circle represents a participant. Solid lines joining filled circles indicate the changes in the level of IFN-γ over 12 months in each participant. The broken horizontal line represents the cut-off for classifying participants as positive (greater or equal to the cut-off) and negative (less than the cut-off). Almost all converters had IFN-γ levels much higher than the cut-off value. (DOC 162 KB)

Below are the links to the authors’ original submitted files for images.

Authors’ original file for figure 1

Authors’ original file for figure 2

Authors’ original file for figure 3
