# Supplementary material for: QuantiFERON-TB Gold In-Tube test conversions and reversions among tuberculosis patients and their household contacts in Addis Ababa: a one year follow-up study
Source: BMC Infect Dis. 2014 Dec 3;14:654. doi: 10.1186/s12879-014-0654-5 (PMC4264256; doi:10.1186/s12879-014-0654-5)
Supplement: Supplementary file 1 — Additional file 1: Levels of IFN-γ at baseline and 12 months later among 24 contacts with baseline QFT-GIT negative results. Wilcoxon matched-pairs signed rank test was used to analyze the data. Each filled circle represents a participant. Solid lines joining filled circles indicate the changes in the level of IFN-γ over 12 months in each participant. The broken horizontal line represents the cut-off for classifying participants as positive (greater or equal to the cut-off) and negative (less than the cut-off). Almost all converters had IFN-γ levels much higher than the cut-off value. (DOC 162 KB) [file 12879_2014_654_MOESM1_ESM.doc]

Additional file 1. Levels of IFN-γ at baseline and 12 months later among 24 QFT negative contacts.
