# Supplementary figures and images for: QuantiFERON-TB Gold In-Tube test conversions and reversions among tuberculosis patients and their household contacts in Addis Ababa: a one year follow-up study
Source: BMC Infect Dis. 2014 Dec 3;14:654. doi: 10.1186/s12879-014-0654-5 (PMC4264256; doi:10.1186/s12879-014-0654-5)

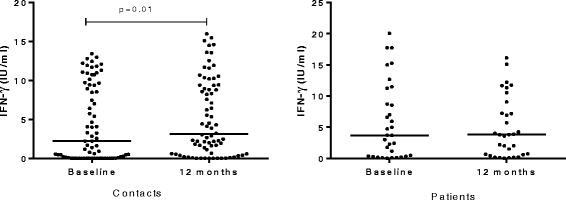

Supplement: Supplementary file 2 — Authors’ original file for figure 1 [file 12879_2014_654_MOESM2_ESM.gif]

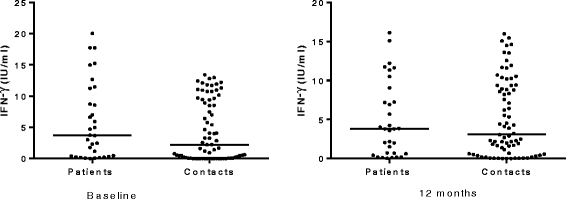

Supplement: Supplementary file 3 — Authors’ original file for figure 2 [file 12879_2014_654_MOESM3_ESM.gif]
