# Supplementary material for: QuantiFERON-TB Gold In-Tube test conversions and reversions among tuberculosis patients and their household contacts in Addis Ababa: a one year follow-up study
Source: BMC Infect Dis. 2014 Dec 3;14:654. doi: 10.1186/s12879-014-0654-5 (PMC4264256; doi:10.1186/s12879-014-0654-5)
Supplement: Supplementary file 4 — Authors’ original file for figure 3 [file 12879_2014_654_MOESM4_ESM.doc]

Figure 3: Levels of IFN-γ at baseline and 12 months later among 24 QFT negative contacts.
